# Clinical Outcomes, Return to Sport and Psychological Readiness After Arthroscopic Bankart Repair Using Knotless All-Suture Anchors at a Minimum 2Year Follow-up

**DOI:** 10.1007/s00402-026-06295-3

**Published:** 2026-05-13

**Authors:** Peter Rab, Marco-Christopher Rupp, Alexander Pfarrmaier, Romed P. Vieider, Igor J. Shirinskiy, Bastian Scheiderer, Sebastian Siebenlist, Lucca Lacheta

**Affiliations:** 1 Department of Sports Orthopaedics, Technical University of Munich, Munich, Germany; 2 Shoulder and Elbow Unit, Department of Orthopedic Surgery, Onze Lieve Vrouwe Gasthuis, Amsterdam, Netherlands

**Keywords:** Shoulder instability, Arthroscopic Bankart Repair, All-Suture Anchor, Knotless, Return to Sports, Psychological Readiness, SI-RSI Scale

## Abstract

**Purpose:**

To report the clinical outcomes, return to sport (RTS) and psychological readiness of patients who underwent arthroscopic Bankart repair with knotless all-suture anchors with a minimum follow-up of 2 years.

**Methods:**

In this retrospective case series, consecutive patients who underwent primary arthroscopic Bankart repair using knotless all-suture anchors between 08/2019 and 07/2022 were included. Patient-reported outcomes were assessed using the Western Ontario Shoulder Instability Index (WOSI), American Shoulder and Elbow Surgeons (ASES) score, Disabilities of the Arm, Shoulder and Hand questionnaire (DASH), Shoulder Instability-Return to Sport after Injury (SI-RSI) scale, subjective shoulder value (SSV), and the visual analogue scale (VAS) for pain. Patient satisfaction, RTS, return to preinjury level of sport, instability recurrence and revisions were recorded. Receiver operating characteristic (ROC) curve was calculated to assess the discriminative performance of the SI-RSI scale, and the Youden’s index was employed to determine the optimal cutoff for prediction of return to preoperative level of sports.

**Results:**

Of 57 patients eligible for inclusion, 46 patients (11.1% female, 28.7 ± 6.8 years at surgery) were available at a follow-up of 2.9 [2.3–3.4] years. Three patients (6.5%) reported a redislocation, one patient (2.2%) underwent a revision and was excluded from analysis. At final follow-up, an ASES score of 98 (92–100), a DASH score of 2.5 (0-6.7), a WOSI of 11 (3.3–18), an SSV of 93 (85–97) along with low levels of pain were reported. A total of 43 (97.7%) of patients reporting preoperative activity (*n* = 44) achieved RTS, with 20 patients (45.5%) who had returned to preoperative level of sports. Patients achieving return to preoperative level of sports had a significantly higher SI-RSI scale (89 [83–94]) than those who did not (61 [50–81], *p* < 0.001). The SI-RSI showed high discriminative performance for return to preoperative level of sports (area under ROC curve: 0.84 [95%CI 0.73–0.97]) with an optimal cutoff of 80 (Youden’s index: 0.597).

**Conclusion:**

At short-term follow-up, Bankart repair using knotless all-suture anchors demonstrated favorable patient-reported outcomes and low redislocation rates. Patients who did not return to their preinjury level of sport exhibited significantly lower psychological readiness. The SI-RSI exhibited high discriminative performance in predicting return to preoperative level of sports, with an optimal cutoff value of 80.

**Level of evidence:**

IV – Retrospective case series.

## Introduction

Anterior shoulder instability is a common condition, particularly prevalent among athletes [1, 2]. Surgical treatment is recommended in cases of recurrent shoulder instability or following a first-time shoulder dislocation with a high risk of recurrence in the presence of risk factors such as young age (< 40 years), contact sports, male sex and hyperlaxity [3, 4]. In the absence of (sub)critical bone loss, Bankart repair is the surgical technique of choice and has evolved from an open to an arthroscopic technique, with considerable advancements in surgical techniques and implant designs over the years [5–7]. In this context, the use of all-suture anchors has increased with the advantage of superior preservation of glenoid bone stock, reduction of the risk of potential complications associated with hard-bodied anchors such as postage stamp fractures, and superior conditions for eventual revision surgery, if required [8–10]. A further advancement in implant design has been the development of tensionable knotless all-suture anchors, which reduce the risk of potential complications associated with knot tying, such as chondral abrasion and knot impingement [11–13]. Although biomechanical analyses have shown similar failure loads and stiffness [11, 13], there is limited data on clinical outcomes and particularly RTS [14, 15].

In addition to patient-specific characteristics and surgical technique, psychological factors can influence the ability to RTS after shoulder instability surgery, yet traditional screening tools may not accurately identify these factors [16–18]. Recent research has established the Shoulder Instability-Return to Sport after Injury (SI-RSI) scale [19, 20]. This scale has been reported as a valid scoring system with a high predictive value in identifying patients ready to return to sport in a cohort of patients undergoing various shoulder stabilization procedures [19, 20], and yet remains to be validated for patients undergoing arthroscopic Bankart repair. Furthermore, with only one study reporting a cutoff of 55 points to differentiate between patients who can and cannot return to sports, which includes patients undergoing bone block procedures [20], data on the cutoff for SI-RSI for return to preinjury level of sports remains limited and yet to be determined for patients undergoing arthroscopic soft tissue stabilization.

The primary aim of this study was to evaluate the clinical outcome of arthroscopic knotless all-suture anchor Bankart repair in patients with anterior shoulder instability with a minimum follow-up of 2 years. It was hypothesized that this procedure would provide satisfactory patient-reported outcomes and acceptable redislocation and revision rates. The secondary aim of this study was to validate the discriminative ability of SI-RSI scale in patients undergoing Bankart repair to determine the threshold for the ability to confidently predict return to preoperative level of sports after arthroscopic Bankart repair. It was hypothesized that the SI-RSI scale would demonstrate high discriminative performance and effectively predict return to preoperative level of sports, with an identified cutoff showing satisfactory sensitivity and specificity.

## Materials and methods

This retrospective monocentric outcome study was conducted in accordance with the Declaration of Helsinki and approved by the Ethics Committee of the Technical University of Munich (Reference: 2023-477-S-SB). Patients with first-time or recurrent anterior shoulder dislocation who underwent arthroscopic Bankart repair with knotless all-suture anchors between 08/2019 and 07/2022 with a minimum follow-up of 24 months were included and contacted exclusively for this study. Patients with posterior or multidirectional instability, fractures of the anterior glenoid rim, and patients younger than 18 years were excluded. In addition, previous shoulder surgery and concomitant procedures other than long head of the biceps tendon (LHBT) tenodesis or superior labrum anterior to posterior (SLAP) repair were exclusion criteria. Chart review was performed to identify patient demographics such as age at surgery, gender, BMI, American Society of Anesthesiologists physical status classification, worker’s compensation status, smoking status, alcohol consumption, and the etiology of instability, categorized as either primary or chronic. All patients provided written informed consent.

### Patient selection

Surgery was indicated in patients with history of a confirmed anterior shoulder dislocation and symptomatic instability as assessed by the anterior shoulder apprehension test in varying degrees of abduction, relocation release test and anterior drawer according to Gerber [21, 22]. The presence of shoulder hyperlaxity was assessed with the Gagey test as well as the Coudane-Walch test [4, 23, 24]. A preoperative MRI was obtained in all cases. In case of any suspected glenoid bone loss, a computed tomography scan was performed and the glenoid bone loss was assessed using a best-fit circle method [4, 25]. Patients with a glenoid bone loss higher than 13% [24, 26] or an off-track Hill-Sachs defect were excluded from this study. Finally, the presence of specific labral lesion configurations such as Perthes or ALPSA lesions was evaluated and addressed accordingly intraoperatively.

### Surgical technique

Surgery was performed under general anesthesia in the beach chair or lateral decubitus position as reported previously [12, 24]. A standard posterior viewing portal and two anterior working portals were placed. After mobilization of the capsulolabral complex, the amount of capsular shift was determined based on the severity of instability, the degree of capsular laxity, and the amount of tissue mobilization. The first knotless all-suture anchor (FiberTak, Arthrex Inc., FL, USA) was placed at the 5:00 o’clock position in a right shoulder. The repair suture was threaded through the capsulolabral complex inferior to the anchor position including the anterior inferior glenohumeral ligament (IGHL) complex, forming a simple stitch pattern, thereby tightening the axillary pouch and creating a superior capsule shift. The self-locking mechanism was buried into the bone tunnel by passing the repair suture through the looped end of the shuttle suture. The capsulolabral complex was positioned using a grasper and tightened using the self-locking mechanism. The tension was adjusted and the sutures cut flush. The number of anchors used was based on the size of the labral tear, with a minimum of three and preferably four anchors being placed. For the right shoulder, anchors were typically placed at the 5:00, 4:00, 3:00, and 2:00 o’clock positions. In case of glenohumeral hyperlaxitiy, an additional anchor was placed at the postero-inferior 7:00 o’clock position with capsular shift of the posterior IGHL complex to symmetrically reduce labral volume [24].

### Postoperative rehabilitation

Following surgery, the arm was protected in a sling for four weeks and external rotation was limited to 0°. Physiotherapy was commenced with the focus on gentle passive motion, pain relief, and the reduction of swelling. By the fifth week, patients were permitted to have full, unrestricted passive and active range of motion of the shoulder. Strengthening exercises, beginning with isometrics and progressing to closed-chain exercises, were introduced at this time. Open chain strengthening exercises were implemented at 6 to 7 weeks postoperatively, and patients were cleared to return to full activity at 4 months.

### Outcome measurements

Preoperative patient-reported outcome measurements (PROM) were recorded prospectively, including the American Shoulder and Elbow Surgeons (ASES) score, the Disabilities of the Arm, Shoulder and Hand (DASH) questionnaire, and the visual analogue scale (VAS) for pain. At final follow-up, patients were contacted and ASES score, DASH, Western Ontario Shoulder Instability Index (WOSI), subjective shoulder value (SSV), the SI-RSI scale and VAS for pain were obtained. RTS was defined as return to the patient’s preinjury sports discipline at any level, and return to preoperative level of sports was defined as return to the patient’s preinjury level of sports. Patient satisfaction was assessed using a five-point Likert scale (5, very satisfied; 4, satisfied; 3, neither satisfied nor unsatisfied; 2, unsatisfied; 1, very unsatisfied) [27]. In addition, the qualitative change in working ability was measured using an ordinal scale (“improved”, “equal to preoperative state”, or “deteriorated”). If patients reported a reduction in working ability, they were asked to specify whether this was attributable to the operated shoulder or other factors. Finally, postoperative redislocations, subluxations, subjective apprehension during overhead activities, and revision rates as well as revision procedures were recorded.

### Statistical analysis

Statistical analysis was performed using R version 4.3.2 (R Foundation for Statistical Computing, Vienna, Austria) and RStudio (Posit Software, PBC, USA). The normality of the distribution was evaluated using the Shapiro-Wilk test and histograms. Continuous variables that were normally distributed are presented as mean ± standard deviation, while those that were not normally distributed are displayed as median and [interquartile range (IQR)]. Categorical variables are presented as counts and percentages. Pre- and postoperative outcome measurements were compared using paired t-tests or Wilcoxon tests, depending on the distribution. Spearman’s rank-order correlation was employed to evaluate the correlation between the SI-RSI scale and patient-reported outcome, including ASES, WOSI and DASH scores, SSV, VAS for pain, as well as demographic factors such as patient age at the time of surgery, body-mass index (BMI), and follow-up time. Correlation coefficients were graded according to Dancey and Reidy (±0.1 to ±0.3: weak, ± 0.4 to ±0.6: moderate, ± 0.7 to ±0.9: strong, ± 1: perfect) [28]. To assess the discriminative performance of the SI-RSI scale for determining return to preoperative level of sports, a receiver operating characteristic (ROC) analysis was used and the area under the curve was assessed using the ‘cutpointr’ package [29]. The Youden’s index with the highest discriminative power was calculated to identify the optimal cutoff for distinguishing between return and non-return to the preinjury level, assigning equal weight to sensitivity and specificity. Further, a p-value of less than 0.05 was considered statistically significant, and all tests were two-tailed. A sample size calculation was performed with the difference in SI-RSI scale between patients with and without return to preoperative level of sports using G*Power (G*Power 3.1, Düsseldorf, Germany) [30]. Based on previous studies [19, 20], the effect size was set to 0.85, using a two-tailed test, and the significance level was set to 0.05. A minimum of 48 observations was determined to achieve a power of 0.8.

## Results

Between 08/2019 and 07/2022, 57 shoulders in 57 patients were retrospectively identified. Of these, 11 patients (19.3%) were lost to follow-up, resulting in 46 patients (5 female, 11.1%) available for follow-up after a median of 2.9 [2.3–3.4] years (response rate: 80.7%). The majority of cases (*n* = 25, 55.6%) were first-time anterior shoulder dislocations (Table 1). Three patients (6.5%; 2 patients with primary anterior instability and 1 patient with chronic anterior instability) reported a redislocation at a median follow-up of 32 [10–34] months postoperatively. All cases were attributed to sustained trauma during sporting activities. Of these, one patient received an autologous glenoid bone grafting procedure 17 months after the index procedure. This patient was excluded from analysis of patient-reported outcome to avoid confounding the results with the effects of a subsequent surgery with an inherently different surgical technique. The remaining two patients opted for non-surgical treatment due to the lack of apprehension and instability during daily activities and sports. No patient reported subluxations during daily or athletic activities.

**Table 1:** Study cohort characteristics

Study cohort characteristics (*n* = 45)	
Follow-up, months [IQR]	34.5 [28.0–41.3]
Female, n (%)	5 (11.1)
Age at surgery, years ± SD	28.7 ± 6.8
Etiology	
First-time dislocation, n (%)	25 (55.6)
Chronic shoulder instability, n (%)	20 (44.4)
Concomitant hyperlaxity, n (%)	23 (51.1)
Worker’s compensation, n (%)	2 (4.4)
BMI, mean ± SD	23.9 ± 2.8
Smoking status, n (%)	10 (22.2)
Alcohol consumption, n (%)	17 (37.8)
ASA, n (%)	
I	33 (73.3)
II	12 (26.7)
Number of anterior anchors placed, n (%)	
Three	38 (84.4)
Four	7 (15.6)
Posteroinferior anchor placed, n (%)	23 (51.1)
Concomitant procedures, n (%)	6 (13.3)
LHBT tenodesis	3 (6.7)
SLAP repair	3 (6.7)

*ASA* American Society of Anesthesiologists physical status classification; *BMI* Body mass index; *IQR* Interquartile Range; *LHBT* Long head of the biceps; *SD* Standard Deviation; *SLAP* Superior labrum anterior to posterior

### Clinical and functional outcome

Preoperative and postoperative PROMs, pain levels and patient satisfaction are detailed in Table 2. The overall SI-RSI was 80.8 [59.9–89.2]. Compared to the preoperative status, significant improvements in DASH (*p* < 0.001), ASES Score (*p* < 0.001) and VAS during shoulder motion (*p* < 0.001) were observed. At final follow-up, 42 patients (93%) were either “very satisfied” or “satisfied”. Postoperative subjective apprehension was reported by 16 patients (35.6%; 9 patients with primary anterior instability and 7 patients with chronic anterior instability) during overhead activities, whereas no patients reported experiencing apprehension during activities of daily life. A total of 5 patients (11.1%) reported a postoperative reduction in their physical workload. Of these, only 2 (40%) attributed the reduction to complaints related to the operated shoulder, while 3 (60%) cited other reasons for the decrease in working ability. No significant differences were observed between patients with a recreational sports level and higher regarding the WOSI (*p* = 0.60), ASES score (*p* = 0.79), DASH score (*p* = 0.67), SSV (*p* = 0.93), and the SI-RSI scale (*p* = 0.19). Furthermore, no significant differences were reported between patients who received an additional postero-inferior anchor for hyperlaxity and those who did not, in terms of the WOSI (*p* = 0.50), ASES score (*p* = 0.49), DASH score (*p* = 0.71), SSV (*p* = 0.20), or SI-RSI scale (*p* = 0.93).

**Table 2:** Clinical and functional outcome parameters preoperatively and at final follow-up

Measurement parameters		Preoperative	At a final follow-up	*p*-value
DASH, median [IQR]		28 [18–43]	2.5 [0–6.7]	**< 0.001**
ASES Score, median [IQR]		63 [45–80]	98 [92–100]	**< 0.001**
VAS at rest, median [IQR]			0 [0–0]	
VAS at motion, median [IQR]		2.0 [1.0–3.3]	1.0 [0–1.5]	**< 0.001**
WOSI, median [IQR]			11 [3.3–18]	
SI-RSI, median [IQR]			80.8 [59.9–89.2]	
SSV, median [IQR]			93 [85–97]	
Patient satisfaction, n (%)				
very satisfied		27 (60)		
satisfied		15 (33.3)		
neither satisfied		2 (4.4)		
nor unsatisfied				
n.a.		1 (2.2)		

*ASES* American Shoulder and Elbow Surgeons Score; *IQR* Interquartile range; *n.a.* not answered; *VAS* Visual Analog Scale; *SI-RSI* Shoulder Instability-Return to Sport after Injury; *SSV* Subjective Shoulder Value; *WOSI* Western Ontario Shoulder Instability Index. Significant p-values are bolded

### Return to sport

Out of 44 preoperatively active patients, a total of 43 (97.7%) achieved RTS. In contrast, only 20 patients (45.5%) returned to their preoperative level of sports (Table 3). The most frequent reasons for reduced postoperative sporting ability included fear of reinjury (*n* = 11; 46%), issues attributed to the operated shoulder (*n* = 9; 38%), other personal factors (*n* = 6; 25%), and complaints related to other joints (*n* = 2; 8%; reasons not mutually exclusive). Patients who had returned to preoperative level of sports showed a significantly higher SI-RSI scale (88.8 [82.8–94.4]) compared to patients who did not reach their preoperative sporting level (61.0 [50.2–81.4], *p* < 0.001). In contrast, no significant difference in SI-RSI scale was observed between first-time or recurrent shoulder dislocation (*p* = 0.6) or between female and male patients (*p* = 0.32). Moreover, no significant difference in SI-RSI scale was observed between patients who did not return to preoperative level of sports due to issues attributed to the operated shoulder and patients citing other reasons for non-return (*p* = 0.65).

**Table 3:** Return to sports, return to preinjury level of sports and postoperative athletic participation in patients who preoperatively participated in athletic activity (*n* = 44, 97.7%)

Measurement parameters		*n* = 44
Return to sport, n (%)		43 (97.7%)
Patients with primary anterior instability, n (%)		23 (53.5%)
Patients with chronic anterior instability, n (%)		20 (46.5%)
Return to preinjury level of sports, n (%)		20 (45.5%)
Patients with primary anterior instability, n (%)		11 (55.0%)
Patients with chronic anterior instability, n (%)		9 (45.0%)
Sports participation, n (%)		
Overhead		29 (65.9)
Contact sports		4 (9.1)
Winter sports		21 (47.7)
Low-impact sports		40 (90.9)
No sports		1 (2.3)
Sports participation level, n (%)		
Recreational		24 (54.5)
Competitive		20 (45.5)
Professional		0 (0)
Hours per week participating in sports, median [IQR]		5.0 [3.8–6.0]

**Fig. 1:**
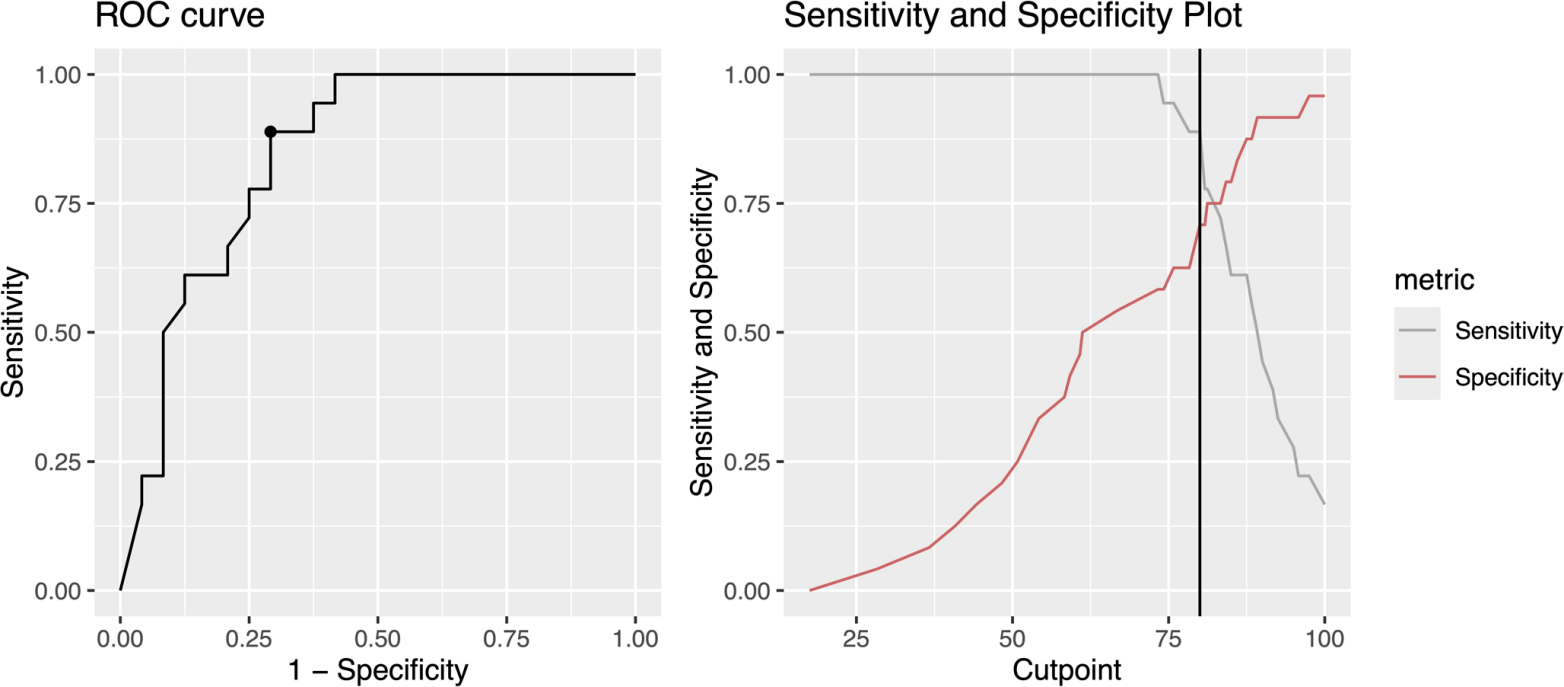
Receiver operating characteristic (ROC) curve and sensitivity and specificity plot for the SI-RSI scale and its discrimination between return to preinjury level of sport vs. no return to preinjury level of sport. The area under the curve is 0.84

A strong correlation between the SI-RSI scale and WOSI (ρ [rho] = −0.84, *p* < 0.001), and a moderate correlation with DASH (ρ = −0.52, *p* = 0.002), ASES score (ρ = 0.60, *p* < 0.001), and VAS at motion (ρ = −0.43, *p* = 0.003) was observed. No correlation with VAS at rest (ρ = −0.23, *p* = 0.13), SSV (ρ = 0.04, *p* = 0.82), BMI (ρ = 0.05, *p* = 0.72), age at the time of surgery (ρ = 0.18, *p* = 0.24), and follow-up time (ρ = 0.1, *p* = 0.5) was reported. The ROC analysis of the SI-RSI scale for determining return to preoperative level of sports showed an area under the curve (AUC) of 0.84 (95% CI 0.73–0.97), see Fig. 1. At the cutoff point of 80, the Youden’s index was maximized at 0.597, corresponding to a sensitivity of 88.9% and specificity of 70.8%.

## Discussion

The most important findings of this study were a favorable shoulder function, a low dislocation rate and a high rate of RTS after arthroscopic Bankart repair using knotless suture anchors at a median follow-up of 2.9 years. Patients who had returned to preoperative level of sports showed a significantly higher SI-RSI scale compared to patients who did not. Furthermore, a high discriminative capacity of the SI-RSI scale was observed and a cutoff value of ≥ 80 was identified to associate with successful return to preoperative level of sports.

Although there is a paucity of data on clinical outcomes following Bankart repair with knotless all-suture anchors, the favorable patient-reported outcomes, high RTS rate, and low recurrent dislocation rate reported in this study are consistent with the broader literature involving various anchor types [17, 31–36]. More specifically for the use of knotless all-suture anchors, Pearce et al. reported a comparable clinical and functional outcome, as well as a high patient satisfaction rate at 2.6 years postoperatively in 31 patients [15]. However, a higher redislocation rate of 12.9% was observed, and the psychological readiness as well as the exact RTS and return to preoperative level of sports rates were not reported [15]. Despite the proposed advantages of knotless anchors, including superior labral reduction and restoration of glenoid concavity, reduced surgical time, and the elimination of chondral abrasion caused by knot tying [11, 13, 37], no superiority in clinical outcome and recurrent instability rates could be shown to date [34, 38–40]. Furthermore, although several authors emphasize the benefits of all-suture anchors in reducing the risk of postage stamp fractures and osteolysis with a superior preservation of glenoid bone stock [8–10], there remains a scarcity of comparative studies. In addition, while the smaller drill tunnel diameter may contribute to bone stock preservation, Lee et al. observed a significantly greater tunnel diameter as well as tunnel diameter increase with all-suture knotless anchors compared to biodegradable anchors at 1 year postoperatively [9]. Repetitive micromotion of the non-biodegradable suture anchor material is cited as a possible cause of the observed tunnel enlargement [9, 41, 42]. In the biomechanical setting, Lacheta et al. [11] reported an equivalent first failure load and ultimate load of knotless all-suture and knotted all-suture anchors in a biomechanical evaluation, regardless of the stitch configuration. However, no study to date has compared patient-reported outcomes of knotless all-suture anchors with knotted all-suture or biodegradable anchor systems. As a result, there remains a paucity of mid-to-long-term clinical and radiographic data comparing knotless all-suture anchors with conventional anchor systems, highlighting the need for further evidence.

This study reported a RTS rate of 97.7%, with 45.5% of patients returning to preinjury level of sports. While data on RTS following Bankart repair with all-suture knotless anchors remains limited, the findings of this study align with the current literature reporting RTS rates ranging from 61% to 94% after Bankart repair [17, 32, 35, 43]. Conversely, the observed rate of return to play at preinjury level has been lower and less consistent, ranging from 9% to 84% [17, 32, 35, 43], particularly among athletes engaging in overhead sports [32, 35, 44]. In this study, the most common reason for a reduced postoperative sporting ability was fear of reinjury. These findings align with a recent systematic review that identified persistent instability, fear of reinjury, and apprehension as key factors hindering RTS [17]. Psychological factors play a substantial role in postoperative rehabilitation and RTS following shoulder surgery and can impact clinical outcomes, as well as the rate and efficacy of RTS [16, 17, 45, 46]. Nevertheless, it remains challenging to identify patients who may not RTS or return to their preinjury level of sports who may benefit from an intensified postoperative care with traditional screening tools. Despite the successful adaptation and validation of the SI-RSI scale from the ACL-RSI scale, data on a cutoff remains scarce [19, 20, 47]. In this context, Rossi et al. [20] proposed a cutoff of ≥ 55 for predicting RTS, achieving a sensitivity of 77% and a specificity of 100%, although the additional inclusion of patients who underwent open Latarjet procedure limits direct comparability. Due to the excellent RTS rate reported in this study, a statistical analysis of the discriminative performance of the SI-RSI for RTS was not feasible. For return to preoperative level of sports, the SI-RSI scale demonstrated a high discriminative performance (AUC 0.84), with a cutoff value of ≥ 80 yielding a sensitivity of 88.9% and a specificity of 70.8%. Consistent with the findings of the present study, Rossi et al. [20] reported an excellent AUC of 0.96 for the SI-RSI scale in predicting return to preoperative level of sports. However, a higher specificity of 90% for the cutoff was observed compared to the present study [20]. Notably, the same cutoff value of ≥ 55 was applied to both RTS and return to preoperative level of sports, however it seems plausible that successfully returning to preoperative level of sports may require a higher threshold due to its greater psychological and physical demands compared to RTS at a lower level [16–18, 20], as demonstrated in the present study with a cut-off value of ≥ 80. Additionally, differences in study cohorts may contribute to the observed variation. A further difficulty in reporting RTS and return to preoperative level of sports rates is the lack of standardization and clear definitions, which may limit comparability and contribute to heterogeneity in reported rates [48].

In this study, the SI-RSI scale showed a strong correlation with the WOSI, a moderate correlation with DASH and ASES score and VAS at motion, but no correlation with VAS at rest or SSV. Several authors have reported a strong correlation between the SI-RSI scale and WOSI [19, 47], which may be explained by similarities in the constructs of the WOSI emotions and SI-RSI emotions subscores, as demonstrated by Olds et al. [47]. The strong correlation with WOSI raises questions about the additional value of the SI-RSI scale in assessing psychological readiness. However, Olds et al. suggested that the SI-RSI evaluates additional emotions, such as fear of reinjury, nervousness and frustration [19, 47, 49]. Moreover, the discrepancy between physical and psychological recovery observed in athletes [19, 47, 49–52], supported by the weak to no correlation between SI-RSI and SSV and moderate correlation with DASH and ASES score observed in this study, suggests that these patient-reported outcome measures may be insufficient to predict RTS or return to preoperative level of sports. As a result, incorporating the SI-RSI scale into clinical practice may provide additional insights into a patient’s psychological readiness to return to athletic activity.

### Limitations

The present study has several considerable limitations. First, a selection bias as well as a recall bias cannot be ruled out which are inherent to retrospective study designs. Second, in accordance with the primary and secondary study aim, this study focused on PROMs without performing an objective clinical examination or radiologic analysis. Third, as the SI-RSI scale was obtained concurrently with patient-reported outcome, its applicability and extrapolation as a predictive tool may be limited when employed during rehabilitation to identify patients at risk of not returning to sport or preinjury level of sports. Fourth, the inclusion of patients with primary and chronic shoulder instability, as well as concomitant SLAP repair and LHBT tenodesis, may introduce heterogeneity to the patient cohort. Fifth, the subgroup analyses conducted were of an exploratory nature, and as a result, were likely underpowered. Sixth, the lack of a control group does not allow comparison with conventional surgical techniques and prevents direct comparative assessment of the knotless technique against other anchor types. Finally, the short-term follow-up of a minimum of 2 years limits the overall strength of conclusions regarding redislocation rates, which are more relevant during mid- and long-term follow-up.

## Conclusion

At short-term follow-up, Bankart repair using knotless all-suture anchors demonstrated favorable patient-reported outcomes and low redislocation rates. Patients who did not return to their preinjury level of sport exhibited significantly lower psychological readiness. The SI-RSI scale exhibited high discriminative performance in predicting return to preoperative level of sports, with an optimal cutoff value of 80.

## Data Availability

Data is provided within the manuscript or supplementary information files.

## Abbreviations

ACL-RSI: Anterior Cruciate Ligament-Return to Sport After Injury
AUC: Area under the curve
ASES: American Shoulder and Elbow Surgeons
BMI: Body Mass Index
DASH: Disabilities of the Arm, Shoulder and Hand
IQR: Interquartile range
LHBT: Long head of the biceps tendon
PROM: Patient-reported outcome measurement
ROC: Receiver operating characteristic
RTS: Return to sports
SI-RSI: Shoulder Instability-Return to Sport After Injury
SLAP: Superior labrum anterior to posterior
SSV: Subjective Shoulder Value
VAS: Visual Analog Scale
WOSI: Western Ontario Shoulder Instability Index
